# ﻿Review of *Asaphes* Walker, 1834 (Hymenoptera, Chalcidoidea, Asaphesinae) from Xinjiang, China

**DOI:** 10.3897/zookeys.1214.127982

**Published:** 2024-10-01

**Authors:** Qin Li, Tong-You Zhang, Gary A. P. Gibson, Shi-Lei Shan, Hui Xiao

**Affiliations:** 1 College of Life Science and Technology, Xinjiang University, 666 Shengli Road, Tianshan District, Urumqi, Xinjiang, 830046, China Xinjiang University Urumqi China; 2 Xinjiang Key Laboratory of Biological Resources and Genetic Engineering, 666 Shengli Road, Tianshan District, Urumqi, Xinjiang, 830046, China Xinjiang Key Laboratory of Biological Resources and Genetic Engineering Urumqi China; 3 Honorary Research Associate, Agriculture and Agri-Food Canada, Canadian National Collection of Insects, Arachnids and Nematodes, K. W. Neatby Bldg., 960 Carling Avenue, Ottawa, Ontario, K1A0C6, Canada Canadian National Collection of Insects, Arachnids and Nematodes Ottawa Canada; 4 Key Laboratory of Zoological Systematics and Evolution, Institute of Zoology, Chinese Academy of Sciences, Beijing, 100101, China Institute of Zoology, Chinese Academy of Sciences Beijing China

**Keywords:** *Asaphes*, COI, integrative taxonomy, morphometrics, Xinjiang

## Abstract

Four species of the cosmopolitan genus *Asaphes* Walker, 1834 (Hymenoptera: Chalcidoidea: Asaphesinae, family incerta sedis) are recorded from Xinjiang Uyghur Autonomous Region, China, bringing the number of known species in China to eight. In addition to *Asaphessuspensus* (Nees ab Esenbeck), 1834 and *A.vulgaris* Walker, 1834, *A.fuyunis* Li & Zhang, **sp. nov.** is newly described based on females and *A.californicus* Girault, 1917, previously known only from North and South America, is newly recorded from China. These four species are differentiated using an integrative taxonomic approach that includes COI barcode data and morphometrics, and are illustrated using macrophotography. Additionally, the 13 described world species of *Asaphes* are tabulated and females of the eight recognized Chinese species are keyed.

## ﻿Introduction

*Asaphes* Walker, 1834 is one of three genera recognized in Asaphesinae by Burks et al. (2022), the other two being *Hyperimerus* Girault, 1917 and *Coriotela* Burks & Heraty, 2020. Both *Asaphes* and *Hyperimerus* are cosmopolitan, whereas *Coriotela* is an extinct genus described from Eocene Baltic amber (Burks and Heraty 2020). The subfamily was historically treated in the family Pteromalidae (Hymenoptera: Chalcidoidea) as Asaphinae prior to Burks and Heraty (2020) providing the new name Asaphesinae when they discovered Asaphinae was a junior homonym of a trilobite family. Subsequently, Burks et al. (2022) removed Asaphesinae from Pteromalidae and treated it as family *incertae sedis* based on the molecular results of Cruaud et al. (2024). Most species of *Asaphes* are hyperparasitoids of aphids (Hemiptera: Aphididae), parasitizing primary hymenopteran parasitoids, including Aphidiinae (Braconidae), *Trechnites* spp. (Encyrtidae), and *Aphelinus* spp. (Aphelinidae) (Bouček 1988; Gibson and Vikberg 1998; de Boer et al. 2019; Kamel et al. 2020).

Prior to the present study, 12 valid world species of *Asaphes* were known (Noyes 2019), including six species from mainland China: *A.globularis* Xiao & Huang, 2000, *A.oculi* Xiao & Huang, 2000, *A.siciformis* Xiao & Huang, 2000, *A.suspensus* (Nees), 1834, *A.umbilicalis* Xiao & Huang, 2000, and *A.vulgaris* Walker, 1834 (Xiao and Huang 2000). Here, we increase the number of species from China to eight by describing one new species, *A.fuyunis* Li & Zhang, sp. nov., and a new record of *A.californicus* Girault, from Xinjiang Uyghur Autonomous Region of China. *Asaphescalifornicus* was previously reported only from North and South America (Gibson and Vikberg 1998, but our new record is based not only on morphological features using the available keys but also by COI molecular and morphometric evidence. A maximum likelihood (ML) tree by K2P distances based on COI sequences and morphometric evidence are provided to support the presence of the four *Asaphes* species in Xinjiang. Additionally, the 13 described world species of *Asaphes* are tabulated and females of the eight species recorded from China are keyed.

## ﻿Materials and methods

### ﻿Morphological studies

Specimens of the four herein treated species from China were collected by sweeping with a net in Xinjiang Uyghur Autonomous Region, 2020–2022, and preserved in 100% ethanol at -20 °C. All specimens are deposited in the Insect Collection of the College of Life Science and Technology, Urumqi, Xinjiang, China (**ICXU**). The **s**pecimens were air dried from ethanol, glued on triangular cards, and examined with a Nikon SMZ 745T stereomicroscope. Dried, point-mounted specimens of *A.californicus* from North America that were used for comparative studies were obtained from the Canadian National Collection of Insects, Arachnids and Nematodes, Ottawa, ON, Canada (**CNC**), and are deposited in ICXU as voucher specimens. Images were taken with a Nikon DS-Fi3 camera connected to a Nikon SMZ 25 camera stereomicroscope. All images were stacked with NIS-Elements software and arranged in plates using Adobe Photoshop. All specimens were identified using Graham (1969), Kamijo and Takada (1973), Gibson and Vikberg (1998), Xiao and Huang (2000), and Narendran and van Harten (2007).

Morphological terms follow Bouček (1988) and Gibson (1997). Body length excludes the protruding parts of ovipositor sheaths and was measured in millimeters (mm); other measurements are given as ratios. Abbreviations of morphological terms used are:

**ED** shortest distance between the inner margins of the eyes;

**EH** eye height;

**EL** eye length;

**EW** eye width;

**Fu_n_** antennal funicular 1, 2…;

**Gt_n_** gastral tergite 1, 2…;

**HL** head length;

**HW** head width;

**mps** multiporous plate sensilla,

**MV** marginal vein;

**OOL** shortest distance between eye margin and a posterior ocellus;

**PMV** postmarginal vein;

**POL** shortest distance between posterior ocelli;

**SMV** submarginal vein;

**STV** stigmal vein.

### ﻿Morphometrics

Forty morphometric variables of seventeen females (four of *A.californicus*, three of *A.fuyunis*, five of *A.suspensus*, and five of *A.vulgaris*) were included in the morphometrical analysis (Table 2). A Principal Components Analysis of the morphometric data in the ADEGENET package in R (Jombart et al. 2010) was conducted to distinguish the four species.

The following abbreviations are used for structures measured:

**A_n_L** length of anellus 1, 2…;

**CL** length of clava;

**CW** width of clava;

**DL** length of dorsellum;

**DW** width of dorsellum;

**Fu_n_L** length of funicle 1, 2…;

**Fu_n_W** width of funicle 1, 2…;

**FRL** length of frenum;

**FWL** length of fore wing;

**FWW** width of fore wing;

**GL** length of gaster;

**Gt_n_L** length of gastral tergite 1, 2…;

**GW** width of gaster;

**IL** distance between the inner orbits in dorsal view;

**MFL** length of metafemur;

**MFW** width of metafemur;

**ML** length of mesoscutum;

**MTAL** length of metatarsus;

**MTL** length of metatibia;

**MTW** width of metatibia;

**MW** width of mesoscutum;

**PDL** length of pedicel;

**PDW** width of pedicel;

**PFCL** combined length of pedicel and flagellum;

**PL** length of propodeum;

**PRL** length of pronotum;

**PRW** width of pronotum;

**PTL** length of petiole;

**PTW** width of petiole;

**PW** width of propodeum;

**SCPL** length of scape;

**SCPW** width of scape;

**SL** length of scutellum; -

**SW** width of scutellum;

**TA** distance from dorsal margin of torulus to ventral margin of anterior ocellus;

**TC** distance from ventral margin of torulus to apical margin of clypeus;

**TL** length of temple in dorsal view;

**UL** length of uncus of stigmal vein.

Acronyms for specimen depositories are as follows: **CNC**, Canadian National Collection of Insects, Arachnids and Nematodes, Ottawa, ON, Canada; **DZUC**, Department of Zoology, University of Calicut, Calicut, Kerala, India; **HOPE**, Hope Entomological Collection, Oxford, England; **ICXU**, Insect Collection of College of Life Science and Technology, Urumqi, Xinjiang, China; **IZCAS**, Institute of Zoology, Chinese Academy of Sciences, Beijing, China; **MZLU**, Lund Museum of Zoology, Lund, Scania, Sweden; **NHMUK** (formerly BMNH), Natural History Museum, London, England; **USNM**, US National Museum of Natural History, Washington, DC, USA.

### ﻿DNA extraction, mtDNA COI amplification, and sequencing

Genomic DNA was extracted from either from individuals preserved in 100% ethanol at -20 °C (Chinese specimens) or dried, point-mounted specimens (*A.californicus*) through whole body extraction using a DNA extraction kit (TIANamp Genomic DNA Kit, China) following the manufacturer’s protocol. In both processes the mixture of proteinase K and Buffer GA were the same and both were held at a constant 56 °C temperature in a metal bath, but duration of the treatments differed. The specimens in ethanol were treated at for 5 h, whereas the dried, point-mounted specimens were treated for 12 h. PCR reaction mixture of 2 5μL was prepared with the following composition of 2× Taq Mix 12.5 μL, ddH_2_O 5.5 μL, the forward primer 1 μL, the reverse primer 1 μL and DNA template 5 μL. The primers of mtDNA COI sequences for *Asaphes* were designed based on sequences of *Asaphes* and *Hyperimerus*, plus those of *Chlorocytus* Graham, 1956, *Dinarmus* Thomson, 1878 and *Mesopolobus* Westwood, 1833 (Pteromalidae: Pteromalinae) on GenBank (www.ncbi.nlm.nih.gov/Genbank) using the software DNAMAN 9.0.1.116 and SNAPGENE 4.1.9. DNAMAN 9.0.1.116 was used to proofread and analyze the specific single nucleotide polymorphism (SNP) site differences in the COI sequences and design specific primers for *Asaphes* by SNAPGENE 4.1.9. The forward primer and reverse primers, respectively, are: 5′- ACC TGT AAT AAT AGG AGG ATT TGG -3′ and 5′- TAA TAG CTC CCG CTA AAA CTG GT-3′. Thermocycling conditions included an initial denaturing step at 95 °C for 4 min, followed by 42 cycles of 95° for 30 sec, 46° for 30 sec, 72° for 1 min and an additional extension at 72° for 10 min. Amplified products were secession on 1% agarose stained with Nucleic acid dye and visualized using a UV trans-illuminator. PCR products were purified and double stranded products were bidirectionally sequenced by Sangon Biotech.

### ﻿Sequence data and phylogenetic analysis

Sequences from both directions were assembled and edited in BIOEDIT v. 7.0.5.3. The COI data set was chosen for phylogenetic analysis and was aligned using the Alignment (Align by Clustal W) multiple alignment program built in MEGA X with the default alignment parameters. Pairwise nucleotide sequence divergences were calculated using a Kimura 2-parameter (K2P) model of substitution (Kimura 1980) and construct the phylogenetic tree ML (Maximum likelihood) in MEGA X (Alajmi et al. 2020; Malagón-Aldana et al. 2022). The robustness of the node of the phylogenetic tree was estimated from 1,000 bootstrap replicates. Based on genetic distance and phylogenetic tree construction, molecular identification was conducted for further verify the results of morphological identification.

COI sequences of *A.vulgaris*, and Pteromalidae sp. as the out-group, were downloaded from NCBI. The details of the sequences are shown in the Table 1.

**Table 1.** Detailed information about NCBI-downloaded sequences of *A.vulgaris* and Pteromalidae sp.

GenBank accession	Morphospecies	Collectors	Collection site
ON704783.1	*Asaphesvulgaris*	Zhang, X	China, Ningxia, Yinchuan
KY912683.1	*Asaphesvulgaris*	Ye, Z., Vollhardt, I.M.G., Tomanovic, Z. and Traugott, M.	Austria, Tirol, Innsbruck
MT878057.1	Pteromalidae sp.	Woolley, V.C., Tembo, Y. and Ndakidemi, B., et al.	United Kingdom, Greenwich, London

**Table 2.** The main ratios measured for characters of female *Asaphes*.

No.	Ratio character	Number	Ratio character
1	FW/FH	21	PRW/PRL
2	EH/EW	22	MW/ML
3	ED/FW	23	SW/SL
4	TA/TC	24	ML/SL
5	SCPL/SCPW	25	SL/FRL
6	PDL/PDW	26	DW/DL
7	An_2_L/An_1_L	27	PW/PL
8	Fu_1_L/Fu_1_W	28	PTL/PTW
9	Fu_2_L/Fu_2_W	29	FWL/FWW
10	Fu_3_L/Fu_3_W	30	MV/ PMV
11	Fu_4_L/Fu_4_W	31	MV/STV
12	Fu_5_L/Fu_5_W	32	PMV/STV
13	Fu_6_L/Fu_6_W	33	SMV/ MV
14	CL/CW	34	STV/UL
15	PFCL/ FW	35	GL/GW
16	HL/HW	36	Gt_1_L/ Gt_2_L
17	EL/EW	37	FML/FMW
18	EL/TL	38	MTL/MTW
19	POL/OOL	39	FML/ MTL
20	IL/HL	40	MTL/MTAL

## ﻿Results

### ﻿Taxonomy

#### ﻿ Asaphes

Taxon classificationAnimaliaHymenopteraPteromalidae

Walker, 1834

AEA9E9AB-2504-5FCA-A521-6433939D9786

Asaphes Walker, 1834: 151. Type species: Asaphesvulgaris Walker; by monotypy.
Isocratus Förster, 1856: 53, 58. Unnecessary replacement name according to Gahan and Fagan 1923: 18; incorrectly considered as preoccupied by Asaphus Brongniart.
Parectroma Brèthes, 1913: 91. Type species: Parectromahubrichi Brèthes by monotypy. Synonymized by De Santis 1960: 113.

##### Diagnosis.

**Figure 1.**
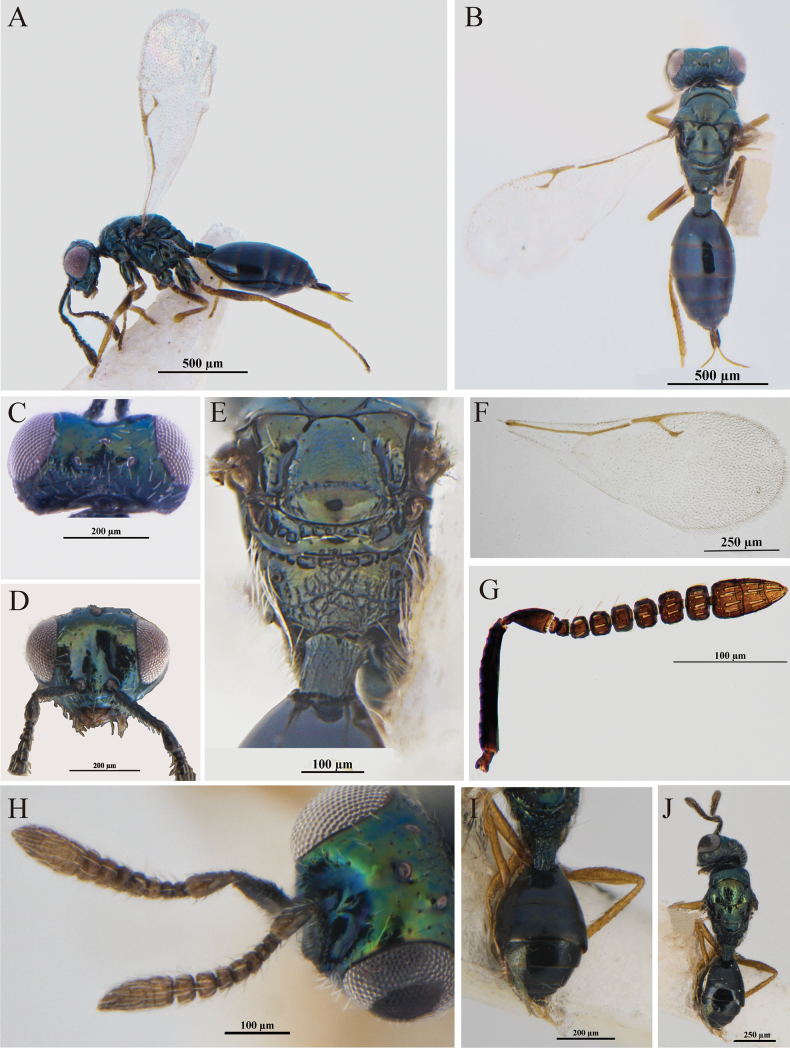
*A.californicus* Girault **A–D** female **A** body, lateral view **B** body, dorsal view **C** head, dorsal view **D** head, frontal view **E** scutellum, propodeum and petiole, dorsal view **F** fore wing **G** antenna **H–J** male **H** antenna **I** petiole and gaster, dorsal view **J** body, dorsal view.

**Figure 2.**
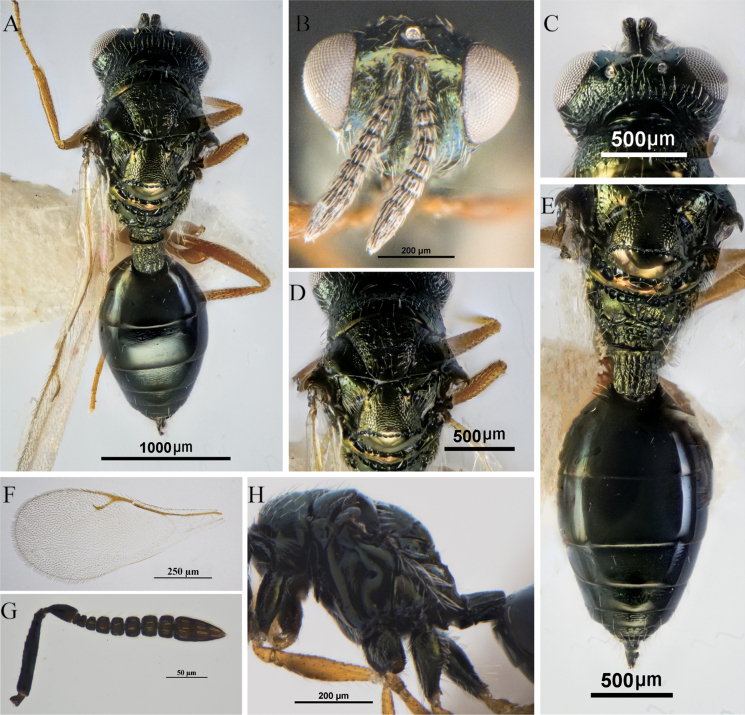
*A.fuyunis* Li & Zhang, sp. nov., holotype, female **A** body, dorsal view **B** head, frontal view **C** head, dorsal view **D** mesosoma; dorsal view **E** propodeum and gaster, dorsal view **F** fore wing **G** antenna **H** prepectus.

**Figure 3.**
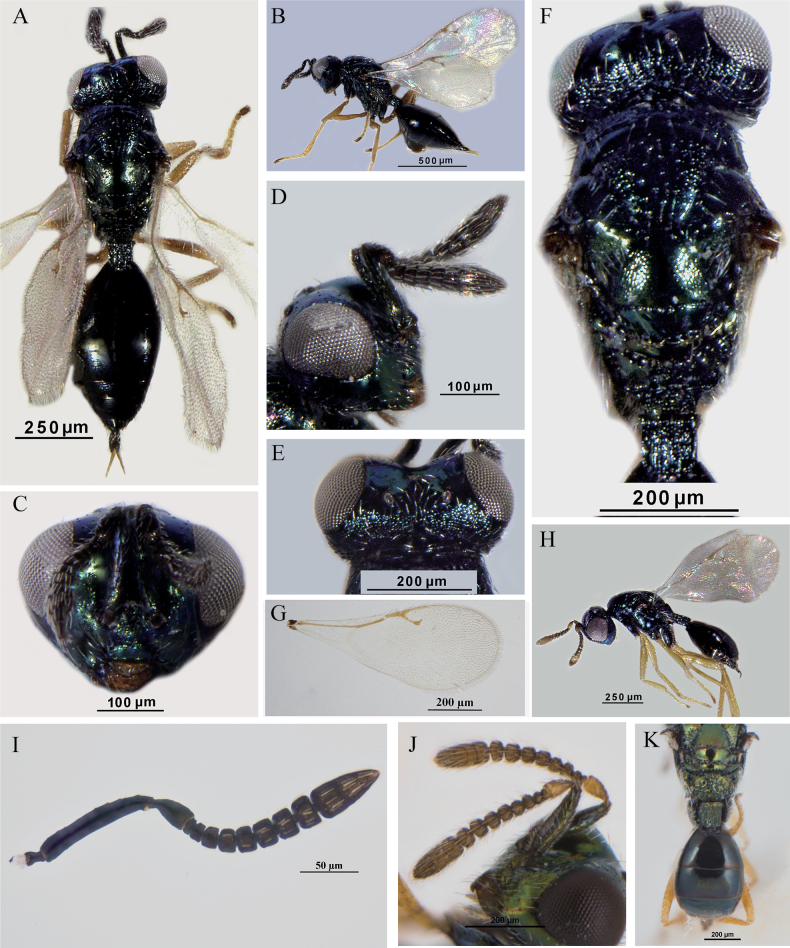
*A.suspensus* (Nees) **A–G, I** female **A** body, dorsal view **B** body, lateral view **C** head, frontal view **D** head, lateral view **E** head, dorsal view **F** head and mesosoma, dorsal view **G** fore wing **I** antenna **H, J, K** male **H** body, lateral view **J** antenna **K** propodeum and gaster, dorsal view.

**Figure 4.**
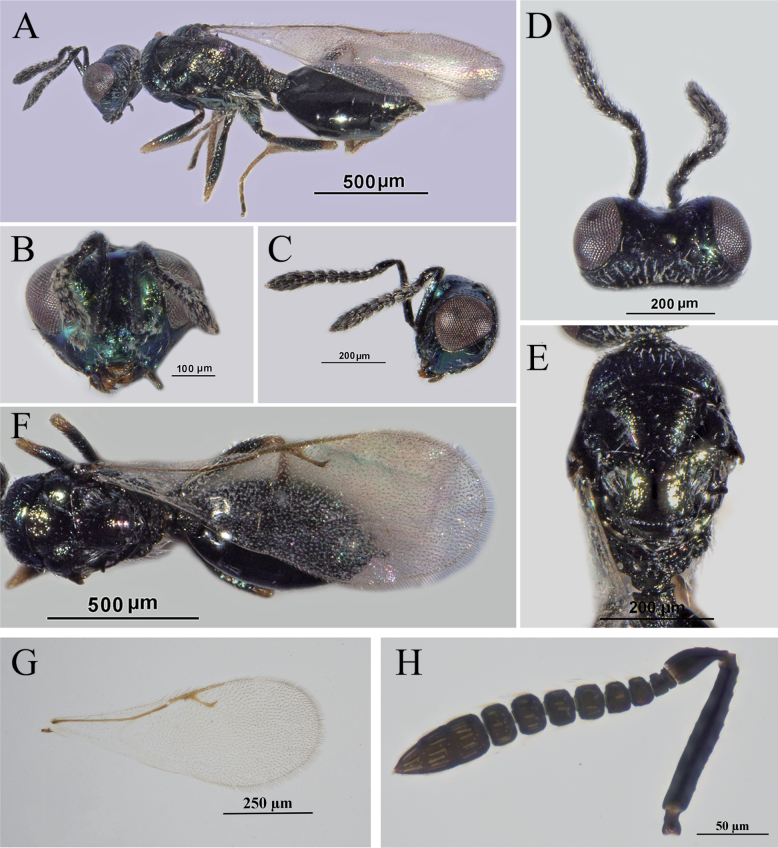
*A.vulgaris* Walker, female **A** body, lateral view **B** head, frontal view **C** head, lateral view **D** head, dorsal view **E** mesosoma, dorsal view **F** mesosoma, metasoma and wing, dorsal view **G** fore wing **H** antenna.

*Asaphes* can be recognized by the following features: head with horseshoe-like occipital carina (Figs 1C, 2C) and with genal carina; antenna 14-segmented including one or two anelli (basal flagellomeres without mps), seven or six funiculars (with mps) and three distinct clavomeres plus tiny apical fourth clavomere (terminal button); torulus distinctly below midline of head near lower margin of eyes, with upper margin slightly above (Figs 1D, 4B) to distinctly below lower ocular line; left mandible bidentate and right mandible tridentate; pronotum transverse-quadrangular, ~ 1/2 as long as mesoscutum and rounded to abruptly angled to neck but without marginal rim (Fig. 1A, B); mesoscutum with compete notauli (Figs 1B, 2A, 3F); marginal vein subequal in length or shorter than stigmal vein (Figs 1F, 2F, 3G, 4G); petiole tubular, divided into dorsal and ventral parts by lateral sulcus and with dorsal surface strongly sculptured, reticulate and/or with irregular longitudinal ribs (Figs 1E, 2E); gaster strongly sclerotized, non-collapsing (Figs 1A, B, I, 2E, 3A, H, K).

##### Comments.

Graham (1990: 200) incorrectly listed *Notopodion* Dahlbom, 1857 as a junior synonym of *Asaphes*, which was followed by Gibson and Vikberg (1998) and Xiao and Huang (2000); rather, *Notopodion* is a synonym of *Podagrion* Spinola, 1811 (Torymidae) (Noyes 2019). Gibson and Vikberg (1998) provide a more comprehensive diagnosis of the genus as well as a subfamily diagnosis as then recognized, which was modified by Burks and Heraty (2020) and Burks et al. (2022). Burks et al (2022) considered the antenna of Asaphenesinae to be 14-segmented, including a small, terminal, fourth clavomere. While we follow their interpretation, because of its size the terminal clavomere, or “terminal button”, is inconspicuous and the antenna superficially appears to be 13-segmetned with three distinct clavomeres (e.g., Fig. 1G, H). Most *Asaphes* species also have two basal flagellomeres without mps and six funiculars with mps, though the antenna of *A.umbilcatus* has only a single strongly transverse anellus and seven funiculars with mps (Xiao and Huang: fig. 6). Narendran and van Harten (2007) described the antennal formula of *A.ecarinatus* as 1: 1: 3: 7: 3 (i.e., 15-segmented), but their line drawing illustration of the flagellum appears to show a single basal flagellomere without mps, six funiculars with mps, and three clavomeres (i.e., 12-segmented). We did not examine type material to clarify these inconsistencies, but almost certainly the described antennal formula is incorrect, and the basal flagellomere likely is so strongly transverse that it is not clearly illustrated in the line drawing so that the antennal formula likely is 1:1:2:6:3, excluding the terminal button. The number of basal flagellomeres lacking mps requires close examination because even though Xiao and Huang (2000) key both *A.suspensus* and *A.vulgaris* as having “at most F1 without sensilla”, the flagellum of both species have two anelli, i.e., lacking mps (Gibson and Vikberg 1998 figs 28, 30). As such, the number of basal flagellomeres without mps for the new species described by Xiao and Huang (2000) requires confirmation, including *A.globularis*, which has the basal four flagellomeres so strongly transverse as to possibly lack mps (Xiao and Huang 2000: fig. 16).

*Asaphes* can be differentiated from other genera classified in Pteromalidae prior to Burks et al. (2022) using such keys as Graham (1969), Bouček and Rasplus (1991), Bouček and Heydon (1997), Xiao and Huang (2000), or Huang and Xiao (2005).

### ﻿Key to Chinese species of *Asaphes* based on females

**Table:** 

1	Temple setose posteriorly; malar space ~ 1/3 length of eye height	***A.oculi* Xiao & Huang, 2000**
–	Temple bare posteriorly; malar space ~ 1/2 length of eye height	**2**
2	Length of flagellum and pedicel combined slightly greater than head width; petiole slightly transvese, 0.8× as long as broad	***A.siciformis* Xiao & Huang, 2000**
–	Length of flagellum and pedicel combined slightly less than head width; petiole at leastquadra-te and usually slightly longer than wide	**3**
3	Mesoscutum with umbilicate punctuation (Xiao and Huang 2000: fig. 7); metacoxa dorsally bare	***A.umbilicalis* Xiao & Huang, 2000**
–	Mesoscutum with shallow engraved reticulation; metacoxa setose dorsally	**4**
4	POL at most 2.2× OOL	***A.globularis* Xiao & Huang, 2000**
–	POL at least 2.3× OOL	**5**
5	Fore wing with speculum distinct (Gibson and Vikberg 1998: figs 68, 70)	**6**
–	Fore wing with speculum absent or indistinct (Gibson and Vikberg 1998: fig. 67)	**7**
6	Head in dorsal view with distinct emargination between inner orbits and temples almost straight (Fig. 4D); hind leg with trochanter and femur similarly infuscate to black (Fig. 4A); fore wing speculum broad basally and narrowing toward stigmal vein (Fig. 4G; Gibson and Vikberg 1998: fig. 70); Gt_1_ slightly longer than Gt_2_	***A.vulgaris* Walker, 1834**
–	Head in dorsal shallowly emarginate between inner orbits and temples curved and rather strongly convergent (Fig. 1C); hind leg with trochanter paler than femur (Fig. 1A); speculum equal in width from parastigma to stigmal vein (Fig. 1F; Gibson and Vikberg 1998: fig. 68); Gt_1_ usually shorter than Gt_2_ (Fig. 1B)	***A.californicus* Girault, 1917**
7	Legs more or less uniformly pale, yellowish to yellowish orange (Fig. 3A, B); stigmal vein 3.3–4.0 × length of uncus	***A.suspensus* (Nees), 1834**
–	Legs reddish brown (Fig. 2A); stigmal vein 2.2–2.6 × length of uncus	***A.fuyunis* Li & Zhang, sp. nov.**

#### ﻿ Asaphes californicus

Taxon classificationAnimaliaHymenopteraPteromalidae

Girault, 1917

3A62E791-198F-5235-A1B2-6EF6FDC7BBD6

Fig. 1

Asaphes californicus Girault, 1917: 1; Gibson and Vikberg 1998: 219–224.

##### Diagnosis.

**Female.** Antenna (Fig. 1G) with pedicel at most 2.0–2.4× as long as wide; clava 2.4–2.5× as long as wide. Head in dorsal view (Fig. 1C) with shallow emargination between inner orbits and temple curved and rather strongly convergent; eye length 3.6–3.8× temple length (Fig. 1A); POL 0.3–0.6× OOL (Fig. 1C). Frenum smooth and shiny except finely carinate laterally (Fig. 1E); metapleuron bare; petiole at least quadrate and usually slightly longer (1.3×) than wide (Fig. 1E). Fore wing (Fig. 1F) with broad speculum. Legs with at least metafemur in part darker than light-colored metatrochanter (Fig. 1A). Gaster (Fig. 1B) 1.4× as long as wide; Gt_1_L usually shorter (0.9×) than Gt_2_L (Fig. 1B).

**Male.** Color pattern similar to female except clava yellowish brown and legs entirely yellow (Fig. 1I). Structure similar to female, with combined length of pedicel and flagellum 0.9× head width; pedicel 2.1× as long as wide; all funicle segments slightly transverse (Fig. 1H) and with few mps; clava 2.4× as long as wide. Petiole (Fig. 1I) 1.4× as long as wide. Gaster (Fig. 1I, J) ovate, 1.8× as long as wide; Gt_1_L almost equal to Gt_2_L.

##### Material examined.

China. Xinjiang • 2♀; Altay Prefecture, Altay City; 47°40'16"N, 88°01'16"E; 710 m; 12 Jul 2020; Qin Li research group • 1♂; Fuyun City; 46°56'10"N, 89°33'51"E; 848 m; 10 Jul 2020; Qin Li research group • 1♀; Hotan Prefecture, Yutian County; 36°90'02"N, 81°40'58"E; 1432 m; 3 Aug 2021; Zhulidezi Aishan research group • 2♀; Ili Kazakh Autonomous Prefecture, Gongliu County; 43°22'60"N, 82°72'09"E; 1137 m; 10 Jul 2021; Qin Li research group.

Canada. Alberta • 1♀1♂; Waterton; 49°06'N, 113°59'W; 1530 m; 11 Jul 1991; H. Goulet. Yukon Territory • 1♀; Alaska Hwy; 60°54'N, 137°09'W; 664 m; 7 Jul 2006; Goulet and Boudreault • 1♂; Alaska Highway E. of Hines Junction; 60°54.062'N, 137°09.791'W; 664 m; sweeping; 7 Jul 2006; Goulet and Boudreault • 1♀; Whitehorse; 60°43'N, 133°03'W; 814 m; 11 Jul 2006; Boudreault and Goulet.

USA. Alaska • 1♀; Wosnesenski Island; 55°12'N, 161°21'W; 11 Jul 2009; Boudreault and Goulet. California • 1♀1♂; Siskiyou County; 2 mi. W. Bartle along McCloud river; 17 Jul.1990; J.D. Pinto.

##### Distribution.

China (Xinjiang) (new country record); Nearctic and Neotropical regions (Noyes 2019).

##### Hosts.

*Asaphescalifornicus* is strictly a hyperparasitoid of aphids through Aphidiinae (Hymenoptera: Braconidae) and Aphelinidae (Hymenoptera) primary parasitoids (Gibson and Vikberg 1998).

##### Comments.

*Asaphescalifornicus* was previously reported only from the Nearctic and Neotropical regions (Gibson and Vikberg 1998). It is reported here from Xinjiang, China for the first time.

#### ﻿ Asaphes fuyunis

Taxon classificationAnimaliaHymenopteraPteromalidae

Li & Zhang sp. nov.

081B5279-0A2F-532F-8339-05A4FB69E0A0

https://zoobank.org/07578F4B-1781-4CF2-918F-B118A2E0C58C

Fig. 2

##### Type material.

***Holotype*** • ♀ (ICXU); China, Xinjiang, Altay Prefecture, Fuyun County, Turhong Township; 47°01'49"N, 89°01'40"E; 1360 m; 11 Jul 2020; Qin Li group. ***Paratypes*** • 3♀; same collection data as holotype.

##### Diagnosis.

Female. Body (Fig. 2A) metallic green, with luster. Head in dorsal shallowly emarginate between inner orbits. Combined length of pedicel and flagellum subequal in width to head. Fore wing hyaline with speculum absent or indistinct; stigmal vein 2.2–2.6 × length of uncus. Legs reddish brown (Fig. 2A). Gt_1_ and Gt_2_ combined are approximately equal to the length of the gaster, with Gt_1_ being longer than Gt_2_ (Fig. 2E).

##### Description.

**Female.** Body (Fig. 2A) length 1.75 mm. Head, mesosoma, and propodeum dark with green and bronze lusters under different angles of light (Fig. 2A–E); antenna dark brown (Fig. 2G) except scape and pedicel concolorous with mesosoma; gaster black or with only slight metallic lusters under some angles of light (Fig. 2A, E); fore wing hyaline with brown venation (Fig. 2F); legs with coxae concolorous with mesosoma, otherwise reddish brown except apical tarsomeres dark brown to black (Fig. 1A).

***Head*** in frontal view (Fig. 2B) transverse-subtriangular, width 1.4× height, with genae distinctly converging ventrally; face with regular, raised reticulation and with dense, white setae; scrobal depression broad and deep, smooth and bare ventrally; clypeus smooth with truncate apical margin. Malar space ~ 1.1× eye height (Fig. 2B). Scape extending to level of vertex (Fig. 2A); pedicel 2.0× as long as wide; funiculars broadly joined, each transverse and with 1 line of mps, with fu_4_ 0.6× as long as wide; clava 1.9× as long as wide; combined length of pedicel and flagellum subequal in width to head. Head in lateral view with eye height 1.7× eye length and 2.5× malar space; malar sulcus absent. Head in dorsal view (Fig. 2C) 2.0× as wide as long; POL 2.6× OOL; gena length 0.5× eye length.

***Mesosoma*** in dorsal view (Fig. 2D) slightly narrower than head width (0.9×); mesosoma compact and convex; pronotum narrower than mesoscutum (0.9×), and 0.6× as long as mesoscutum; collar abruptly margined anteriorly, posterior margin smooth and bare (Fig. 2D); mesoscutum 2.0× as long as broad, equal in length to scutellum; notauli deep and complete; scutellum (Fig. 2D, E) 0.8× as long as broad, with engraved, reticulate sculpture; frenum smooth and shiny, delineated anteriorly by continuous septate frenal line; propodeum (Fig. 2E) 0.8× as long as scutellum, without median carina or plicae, median area with coarse and irregular sculpture, and laterally with dense, whitish, long setae. Mesosoma in lateral view (Fig. 2H) with metapleuron bare. Fore wing (Fig. 2F) densely setose, without distinct speculum; proportions of length of marginal, postmarginal, and stigmal veins 19:24:16; stigmal vein 2.6× as long as uncus. Metacoxa setose both dorsally and ventrally (Fig. 2H).

***Metasoma*** with petiole quadrate, subequal in length and breadth (Fig. 2A, E), dorsally with numerous irregular longitudinal ribs. Gaster (Fig. 2A, E) oval, 1.8× as long as wide; Gt_1_ and Gt_2_ smooth and combined length 0.5× length of gaster, Gt_1_ 1.2× as long Gt_2_.

**Male.** Unknown.

##### Variation.

No significant difference in measurement data.

##### Host.

Unknown.

##### Etymology.

The specific name is derived from the collection locality of its holotype.

##### Distribution.

China (Xinjiang).

##### Comments.

Females of this species have an unusually long malar space for members of *Asaphes*, being ~ 1.1× the height of an eye (Fig. 1B). Leg color is similar to some specimens of *A.suspensus* that have comparatively dark, yellowish orange legs as well as an indistinct fore wing speculum (Fig. 3G), but *A.suspensus* females have the malar space at most ~ 0.7× the height of an eye (Gibson and Vikberg 1998).

#### ﻿ Asaphes suspensus

Taxon classificationAnimaliaHymenopteraPteromalidae

(Nees, 1834)

36A07243-B1EC-5122-91E0-353788A0B457

Fig. 3

Chrysolampus suspensus Nees, 1834: 127; McMullen 1966: 236–239; Graham 1969: 82–83; Gibson and Vikberg 1998: 230–236; Xiao and Huang 2000: 194–195; Huang and Xiao 2005: 273–274.
Chrysolampus altiventris Nees, 1834: 127. Synonymized by Graham 1969: 82.
Pteromalus petioliventris Zetterstedt, 1838: 429. Synonymized by Graham 1969: 82.
Chrysolampus aphidiphagus Ratzeburg, 1844: 181. Synonymized by Graham 1969: 82.
Chrysolampus aphidicola Rondani, 1848: 19–21. Synonymized by Bouček 1974: 244.
Euplectrus lucens Provancher, 1887: 207. Synonymized by Gibson and Vikberg 1998: 231.
Asaphes rufipes Brues, 1908: 160. Synonymized by Gibson and Vikberg 1998: 231.
Megorismus fletcheri Crawford, 1909: 98. Synonymized by Gibson and Vikberg 1998: 231.
Asaphes americana Girault, 1914: 114. Synonymized by Gibson and Vikberg 1998: 231.
Pachycrepoides indicus Bhatnagar, 1952: 160–163. Synonymized by Gibson and Vikberg 1998: 231.
Asaphes sawraji Sharma & Subba Rao, 1959: 181. Synonymized by Bouček et al. 1979: 436.
Pachyneuron uniarticulata Mani & Saraswat, 1974: 96–98. Synonymized by Bouček et al. 1979: 436.

##### Material examined.

China, Xinjiang: Altay Prefecture, Qin Li group • 2♀; Altay City; 47°40'16"N, 88°01'16"E; 710 m; 12 Jul 2020 • 1♀; Fuyun County; 47°01'49"N, 89°53'46"E; 1360 m; 11 Jul 2020 • 1♀; Qinghe County; 46°41'31"N, 90°21'28"E; 1240 m; 10 Jul 2020 • 1♀; Qinghe County; 46°92'88"N, 90°01'62"E; 1427 m; 6 Jul 2021. Bayingol Mongolian Autonomous Prefecture, Hongying Hu group • 1♀1♂; Bohu County; 42°02'63"N, 86°66'48"E; 1053 m; 7 Aug 2010 • 1♀; Yuli County; 41°39'01"N, 86°25'01"E; 871 m; 5 Aug 2010 • 2♀; Bortala Mongolian Autonomous Prefecture, Bole City; 44°87'72"N, 82°14'60"E; 405 m; 30 Jun 2021; Qin Li group. Changji Hui Autonomous Prefecture, Hongying Hu research group • 1♀; Mulei Kazakh Autonomous County; 43°98'32"N, 90°37'70"E; 1219 m; 30 Jul 2012 • 2♀4♂; Qitai County; 43°95'16"N, 89°52'85"E; 833m; 29 Jul 2012 • 1♀; Qitai County; 43°58'27"N, 89°78'10"E; 847 m; 29 Jul 2012. Ili Kazakh Autonomous Prefecture, Hongying Hu research group • 1♀; Huocheng County; 44°06'76"N, 80°85'81"E; 661 m; 22 Jun 2010 • 1♀; Tekes County; 43°23'25"N, 81°84'36"E; 1865 m; 27 Jul 2010. Ili Kazakh Autonomous Prefecture, Qin Li research group • 2♀; Gongliu County; 43°22'60"N, 82°72'09"E; 1137 m; 10 Jul 2021 • 2♀3♂; Huocheng County; 43°94'47"N, 80°87'04"E; 515 m; 5 Jul 2021 • 1♀; Tekes County; 43°22'19"N, 81°88'88"E; 1201 m; 8 Jul 2021 • 2♂; Kashgar Prefecture, Artux City; 39°69'49"N, 76°20'23"E; 1303 m; 22 Jun 2008; Hongying Hu research group • 1♀; Tarbagatay Prefecture, Wusu County; 44°00'43"N, 84°95'34"E; 1908 m; 25 Jul 2013; Hongying Hu research group • 1♂; Urumqi, Tianshan District; 43°77'49"N, 87°62'07"E; 928 m; 17 Apr 2007; Hongying Hu research group.

##### Diagnosis.

**Female.** Antenna (Fig. 3I) with combined length of pedicel and flagellum less than head width; pedicel at most 1.6–1.8× as long as wide; funiculars subquadrate, broadly joined, and each with one line of sensilla; fu_4_ 0.8× as long as broad. Head in dorsal view (Fig. 3E) with shallow emargination between inner orbits; eye length 2.3–2.8× temple length. Frenum smooth and shiny (Fig. 3F); metapleuron bare. Metatibia 6.2–6.8× times as long as wide. Fore wing (Fig. 3G) with speculum indistinct; marginal vein 0.6–0.8× as long as postmarginal vein and stigmal vein 3.3–4.0× as long uncus. Legs (Fig. 3B) more or less uniformly light-colored, yellowish. Petiole (Fig. 3A, F) at least quadrate and usually slightly longer (1.1×) than wide. Gaster 1.9× as long as broad (Fig. 3A).

**Male.** Color pattern brighter than female, and pedicel and flagellum yellowish brown (Fig. 3H, J). Antenna (Fig. 3J) with combined length of pedicel and flagellum 0.9× head width; pedicel 1.8× as long as wide; funicle with all segments slightly transverse; clava 2.3× longer than wide. Petiole (Fig. 3K) 1.2× as long as wide, entirely reticulate with longitudinal carinae. Gaster (Fig. 3H, K) ovate, Gt_1_L 1.1× as long as Gt_2_L. Otherwise similar to female.

##### Distribution.

China (Xinjiang, Beijing, Fujian, Guangdong, Hebei, Heilongjiang, Henan, Hunan, Jilin, Shaanxi, Shanxi, Sichuan, Tibet, Yunnan). Palearctic region and Nearctic region (Noyes 2019).

##### Hosts.

Usually a hyperparasitoid of aphids through Aphidiinae (Hymenoptera: Braconidae) and Aphelinidae (Hymenoptera) primary parasitoids, and rarely parasites *Psylla* Geoffroy, 1762 (Hemiptera: Psyllidae) (Gibson and Vikberg 1998).

##### Comments.

The morphological features of our specimens fit within the limits described for *A.suspensus* by Gibson and Vikberg (1998); they described the length of the pedicel as at most 2× as long as wide, whereas the pedicel of our measured specimens was at most 1.8× as long as wide.

#### ﻿ Asaphes vulgaris

Taxon classificationAnimaliaHymenopteraPteromalidae

Walker, 1834

63DF7A24-E843-5BFE-ACB8-49AE09F157A8

Fig. 4

Asaphes vulgaris Walker, 1834: 152.
Eurytoma aenea Nees, 1834: 42. Synonymized by Graham 1969: 80.
Chrysolampus aeneus Ratzeburg, 1848: 2. Synonymized by Reinhard 1857: 76.
Chrysolampus aphidophila Rondani, 1848: 21–22. Synonymized by Bouček 1974: 244.
Asaphes vulgaris Walker; Morley 1910: 28; Gibson and Vikberg 1998: 236–239; Xiao and Huang 2000: 198; Huang and Xiao 2005: 275–276; Narendran and Harten 2007: 114–116.

##### Material examined.

China, Xinjiang: Altay Prefecture, Qin Li research group • 5♀; Altay City; 47°40'16"N; 88°01'16"E; 710 m; 12 Jul 2020 • 1♀; Fuyun County; 47°01'49"N, 89°53'46"E; 1360 m; 11 Jul 2020 • 1♀; Fuyun County; 47°01'73"N, 89°84'68"E; 1287 m; 22 Jun 2021 • 1♀; Fuyun County; 47°21'60"N, 89°84'43"E; 1141 m; 23 Jun 2021 • 1♀; Qinghe County; 46°43'35"N, 90°04'49"E; 1121 m; 21 Jun 2021 • 1♀; Bayingol Mongolian Autonomous Prefecture, Yuli County; 41°35'11"N, 86°29'45"E; 892 m; 5 Aug 2010; Hongying Hu group. Ili Kazakh Autonomous Prefecture, Qin Li research group • 3♀; Gongliu County; 43°22'60"N, 82°72'09"E; 1137 m; 10 Jul 2021 • 1♀; Huocheng County; 43°94'47"N, 80°87'04"E; 515 m; 5 Jul 2021.

##### Diagnosis.

**Female.** Head in dorsal view (Fig. 4D) with comparatively deep emargination between inner orbits and straight temples; gena length (Fig. 4C) ~ 0.3–0.4× eye length. Antenna (Fig. 4C, H) with each funicular subquadrate, and segments loosely joined to each other. Pronotum (Fig. 4E) 2.3–2.8× wider than long. Hind leg (Fig. 4A) with trochanter and femur similarly infuscate to black; metatibia 7.7–7.8× longer than wide. Fore wing (Fig. 4G) with speculum distinct, broad basally and narrowed toward stigmal vein. Petiole (Fig. 4A) at least quadrate and usually slightly longer (1.1–1.3×) than wide. Gaster (Fig. 4A, F) 1.7–1.9× as long as broad; Gt_1_L slightly longer (1.1×) than Gt_2_L.

**Male.** Unknown.

##### Distribution.

China (Xinjiang, Hebei, Sichuan, Yunnan, Tibet, Ningxia, Guangxi). Worldwide (Noyes 2019).

##### Hosts.

In North America, *A.vulgaris* is a hyperparasitoid of aphids, including *Acyrthosiphonpisum* Harris and *Macrosiphumeuphorbiae* Thomas (Hemiptera: Aphididae) through *Aphidiusnigripes* Ashmead (Hymenoptera: Braconidae) (Gibson and Vikberg 1998).

##### Comments.

Leg color of *A.vulgaris* females is similar to that of *A.californicus* except for trochanter color. Females of *A.vulgaris* have at least the meso- and metatrochanters infuscate to black, similar in color to the respective femora, whereas at least the metatrochanter of *A.californicus* females is mostly yellow, paler than the femur. In our study, gena length is 0.3–0.4× eye length, as described by Huang and Xiao (2005), which differs from the description of 0.5–0.6× eye length given by Gibson and Vikberg (1998); however, this may reflect a somewhat different method of measurement.

### ﻿Morphometrics

The first two principal components (PCA1 and PCA 2) of the PCA analysis recovered 49.3% of the variation in the morphometric and meristic data set (Fig. 6) and loaded most heavily for the ratio of (Fu_4_L/Fu_4_W), (STVL/UL), (PFCL/ FW), (MV/ PMV) along PCA 1 and ratio of (PMV/STV), (IL/HL), (EL/TL), and (CL/CW) along PCA 2 (Table 5). The PCA data strongly support the results of the traditional morphology and molecular data.

### ﻿Molecular results

**Figure 5.**
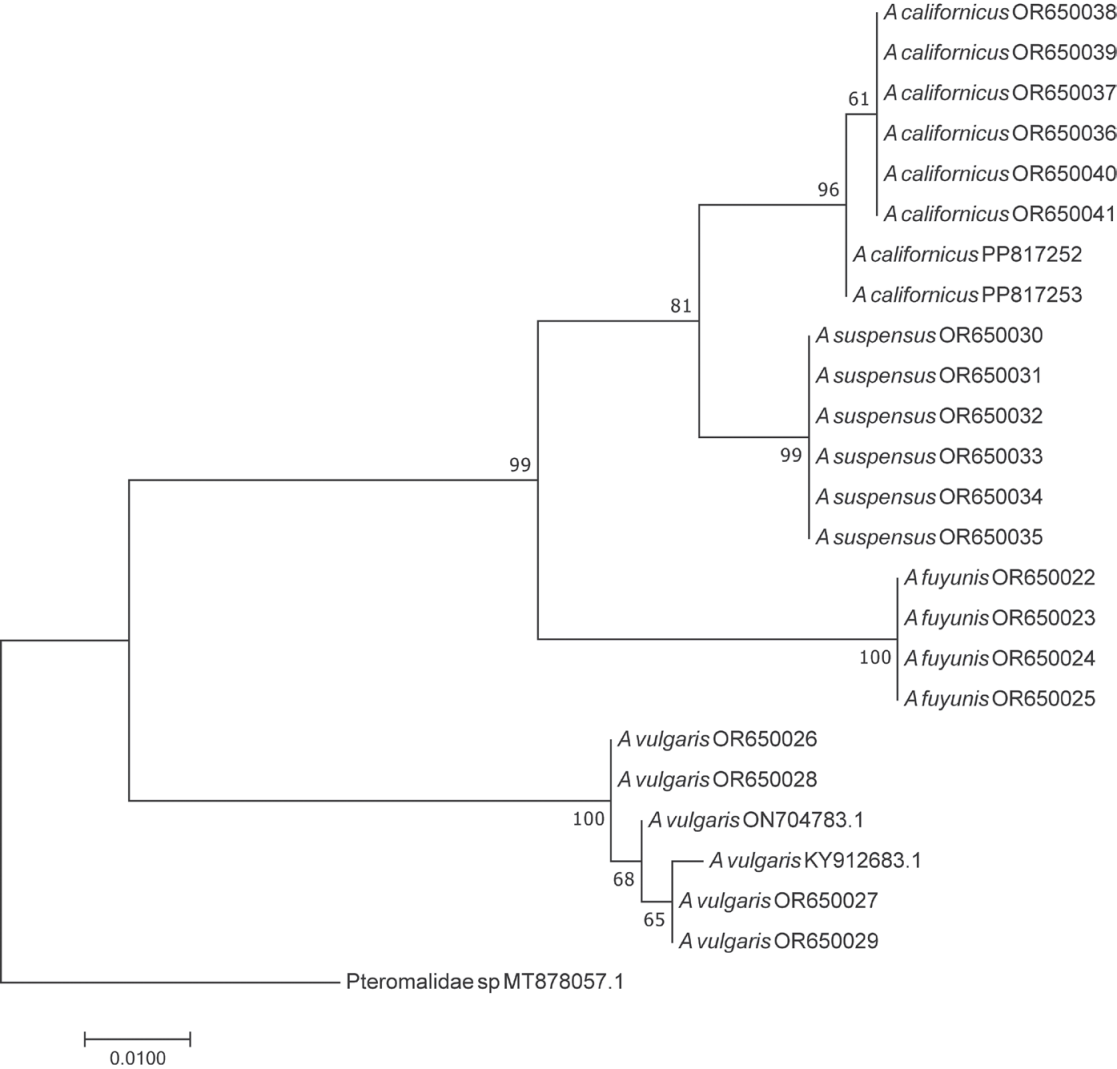
Maximum Likelihood (ML) tree by K2P distances based on COI sequences of *Asaphes*.

We successfully obtained 22 DNA barcode (COI) sequences (see Table 3) from our specimens, in addition to the two sequences of *A.vulgaris* obtained from NCBI (Table 1), which support the presence of four species of *Asaphes* (Fig. 5) in Xinjiang, China. We identify these species as *A.californicus*, *A.fuyunis* sp. nov., *A.suspensus*, and *A.vulgaris*, although molecular evidence from western European specimens of *A.suspensus* is currently lacking to support our identification of this species from China. The two sequences identified as *A.vulgaris* obtained from NCBI do support our identification of *A.vulgaris* from Xinjiang. Genetic Kimura-2 parameter (K2P) distances of the intraspecific and interspecific COI sequences were calculated in MEGA X (Table 4). The results indicate that intraspecific distances are 0.0% to 0.9% and interspecific distances between the four species varied from 2.3% to 11.3%.

**Figure 6.**
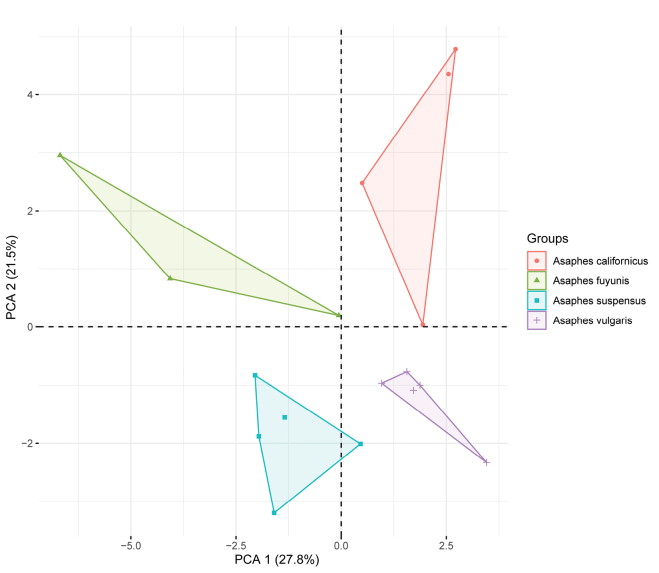
Principal component analysis (PCA) of *Asaphes* species.

**Table 3.** Information on sequenced specimens with GenBank accession of COI.

Specimen number	Morphospecies	GenBank accession number	Sex
1	*Asaphesfuyunis* 1	OR650022	F
2	*Asaphesfuyunis* 2	OR650023	F
3	*Asaphesfuyunis* 3	OR650024	F
4	*Asaphesfuyunis* 4	OR650025	F
5	*Asaphescalifornicus* 1	OR650036	M
6	*Asaphescalifornicus* 2	OR650037	M
7	*Asaphescalifornicus* 3	OR650038	F
8	*Asaphescalifornicus* 4	OR650039	F
9	*Asaphescalifornicus* 5	OR650040	F
10	*Asaphescalifornicus* 6	OR650041	F
11	*Asaphescalifornicus* 7	PP817252	F
12	*Asaphescalifornicus* 8	PP817253	F
13	*Asaphessuspensus* 1	OR650030	F
14	*Asaphessuspensus* 2	OR650031	F
15	*Asaphessuspensus* 3	OR650032	F
16	*Asaphessuspensus* 4	OR650033	M
17	*Asaphessuspensus* 5	OR650034	F
18	*Asaphessuspensus* 6	OR650035	F
19	*Asaphesvulgaris* 1	OR650026	F
20	*Asaphesvulgaris* 2	OR650027	F
21	*Asaphesvulgaris* 3	OR650028	F
22	*Asaphesvulgaris* 4	OR650029	F

**Table 4.** Kimura 2-parameter genetic distances calculated within and between each species of *Asaphes*. **1–4**, *A.fuyunis*, OR650022–OR650025. **5–10**, *A.californicus*, OR650036–OR650041; **11–12**, *A.californicus*, PP817252–PP817253. **13–18**, *A.suspensus*, OR650030–OR650035. **19–22**, *A.vulgaris*, OR650026–OR650029; **23**. *A.vulgaris*, ON704783.1; **24**. *A.vulgaris*, KY912683.1; **25**. MT878057.1Pteromalidae sp.

	1	2	3	4	5	6	7	8	9	10	11	12	13	14	15	16	17	18	19	20	21	22	23	24	25
**1**																									
**2**	0.000																								
**3**	0.000	0.000																							
**4**	0.000	0.000	0.000																						
**5**	0.055	0.055	0.055	0.055																					
**6**	0.055	0.055	0.055	0.055	0.000																				
**7**	0.055	0.055	0.055	0.055	0.000	0.000																			
**8**	0.055	0.055	0.055	0.055	0.000	0.000	0.000																		
**9**	0.055	0.055	0.055	0.055	0.000	0.000	0.000	0.000																	
**10**	0.055	0.055	0.055	0.055	0.000	0.000	0.000	0.000	0.000																
**11**	0.058	0.058	0.058	0.058	0.003	0.003	0.003	0.003	0.003	0.003															
**12**	0.058	0.058	0.058	0.058	0.003	0.003	0.003	0.003	0.003	0.003	0.000														
**13**	0.055	0.055	0.055	0.055	0.026	0.026	0.026	0.026	0.026	0.026	0.023	0.023													
**14**	0.055	0.055	0.055	0.055	0.026	0.026	0.026	0.026	0.026	0.026	0.023	0.023	0.000												
**15**	0.055	0.055	0.055	0.055	0.026	0.026	0.026	0.026	0.026	0.026	0.023	0.023	0.000	0.000											
**16**	0.055	0.055	0.055	0.055	0.026	0.026	0.026	0.026	0.026	0.026	0.023	0.023	0.000	0.000	0.000										
**17**	0.055	0.055	0.055	0.055	0.026	0.026	0.026	0.026	0.026	0.026	0.023	0.023	0.000	0.000	0.000	0.000									
**18**	0.055	0.055	0.055	0.055	0.026	0.026	0.026	0.026	0.026	0.026	0.023	0.023	0.000	0.000	0.000	0.000	0.000								
**19**	0.104	0.104	0.104	0.104	0.099	0.099	0.099	0.099	0.099	0.099	0.096	0.096	0.099	0.099	0.099	0.099	0.099	0.099							
**20**	0.110	0.110	0.110	0.110	0.104	0.104	0.104	0.104	0.104	0.104	0.101	0.101	0.104	0.104	0.104	0.104	0.104	0.104	0.006						
**21**	0.104	0.104	0.104	0.104	0.099	0.099	0.099	0.099	0.099	0.099	0.096	0.096	0.099	0.099	0.099	0.099	0.099	0.099	0.000	0.006					
**22**	0.110	0.110	0.110	0.110	0.104	0.104	0.104	0.104	0.104	0.104	0.101	0.101	0.104	0.104	0.104	0.104	0.104	0.104	0.006	0.000	0.006				
**23**	0.107	0.107	0.107	0.107	0.101	0.101	0.101	0.101	0.101	0.101	0.099	0.099	0.101	0.101	0.101	0.101	0.101	0.101	0.003	0.003	0.003	0.003			
**24**	0.113	0.113	0.113	0.113	0.107	0.107	0.107	0.107	0.107	0.107	0.104	0.104	0.107	0.107	0.107	0.107	0.107	0.107	0.009	0.003	0.009	0.003	0.006		
**25**	0.101	0.101	0.101	0.101	0.099	0.099	0.099	0.099	0.099	0.099	0.096	0.096	0.087	0.087	0.087	0.087	0.087	0.087	0.084	0.090	0.084	0.090	0.087	0.093	

**Table 5.** Summary statistics of the principal component analysis of *Asaphes* species.

No.	Ratio Character	PCA 1	PCA 2	PCA 3	PCA 4	PCA 5
1	FW/FH	0.216603	3.1783295	5.282507068	0.35574012	0.11672155
2	EH/EW	2.87230488	0.01275921	2.403104213	2.77131137	8.66243702
3	ED/FW	0.064479	1.33391248	3.750109321	0.01440452	0.02641105
4	TA/TC	0.38712463	3.84482846	4.943076551	0.01412389	0.86188693
5	SCPL/SCPW	3.77417162	0.17398981	4.917479269	5.42866022	5.15896312
6	PDLL/PDLW	2.48648096	3.47521382	0.865723008	1.62566188	1.32165941
7	An_2_L/An_1_L	0.326642	0.07101507	3.637868218	0.12479640	0.03293327
8	Fu_1_L/Fu_1_W	0.54994757	3.14619133	0.678699525	0.10242453	4.41799084
9	Fu_2_L/Fu_2_W	0.54780303	0.91981684	6.491676859	4.87546358	0.57158251
10	Fu_3_L/Fu_3_W	2.55243666	1.5716729	0.021682635	0.00520423	1.24671401
11	Fu_4_L/Fu_4_W	10.73709122	0.03026482	3.00986227	0.53440516	0.46617328
12	Fu_5_L/Fu_5_W	4.27256446	0.01729463	0.171878122	6.27041051	7.96592789
13	Fu_6_L/Fu_6_W	5.31963583	0.02737012	0.462662143	5.37478061	7.93531917
14	CL/CW	0.02455471	5.55428663	4.020927977	6.27200768	0.73043984
15	PFCL/ FW	6.75428702	1.08591194	2.623988773	2.17895563	0.04085098
16	HL/HW	2.0032471	3.92372966	2.655545541	0.80496354	0.37575628
17	EL/EW	4.0012536	0.28429239	2.524285127	7.50944257	0.554169
18	EL/TL	0.11689347	7.48754868	0.021480775	0.51223903	6.31378767
19	POL/OOL	1.91102438	4.41288002	0.087581884	0.01816490	1.73381626
20	IL/HL	0.00495787	10.4068808	1.652372742	6.98858843	0.03276383
21	PW/PL	5.94539663	2.6230557	1.171565757	1.27406889	0.06924112
22	MW/ML	0.50006149	1.4715376	2.264067963	4.36335336	1.65929375
23	SW/SL	0.43666104	2.93931866	0.469217579	1.78127138	1.49050197
24	ML/SL	1.06564488	4.71355448	0.147326384	1.39131205	8.56569969
25	SL/FREL	0.03131094	0.30214966	0.762210045	14.6648713	5.19235602
26	DW/DL	5.35506306	1.0741069	1.789557847	0.51568116	11.02661261
27	PW/PL	2.01239276	1.79406094	9.730208122	0.06695963	0.157202
28	PTL/PTW	1.80838085	3.7644288	0.729981654	0.35903363	0.0955859
29	FWL/FWW	0.17121849	3.35181891	0.603319812	0.03354785	0.24197932
30	MV/ PMV	6.6232683	2.32960968	0.003427697	1.59581658	0.38893584
31	MV/STV	4.92799266	1.5431306	1.133819339	0.99042593	1.63080091
32	PMV/STV	0.13428142	13.4269751	0.456237813	0.68635732	0.15941343
33	SMV/ MV	3.59396951	0.25035778	2.226292291	6.41494374	0.27368141
34	STV/UL	7.66254386	0.81944172	0.313900134	4.92683749	1.32236046
35	GL/GW	0.01600313	0.43491547	9.055025395	0.07733081	2.85458356
36	Gt_1_L/ Gt_2_L	5.47504516	2.61689068	1.348141421	1.78054706	0.07476514
37	FML/FMW	0.15372799	1.95444963	5.602996723	5.19072632	3.09035096
38	MTL/MTW	2.11187438	3.25304848	4.255962542	0.86010845	1.1979344
39	FML/ MTL	1.70215655	0.36189028	7.626523209	0.74815528	2.27170915
40	MTL/MTAL	1.3495039	0.01706981	0.087706254	0.49690272	9.67068846

## ﻿Discussion

Individuals of *A.fuyunis*, *A.californicus*, *A.suspensus*, and *A.vulgaris* can be difficult to distinguish using traditional morphological features because of multiple variable characteristics, including the depth of the emargination between the inner orbits in dorsal view and leg color. Gibson and Vikberg (1998) described the emargination between the inner orbits of *A.vulgaris* as “relatively deeply” concave, and “relatively shallowly” concave in *A.suspensus* and *A.californicus* (cf. Figs 1C, 3E with 4D). Because this is a relative feature that differs somewhat depending on angle of view, it can be difficult to assess accurately. Gibson and Vikberg (1998) also reported that the trochanters and trochantelli of female *A.californicus* were almost always uniformly yellowish to yellowish brown, paler than the black meso- and metafemora, which matches our specimens from China (Fig. 1A), although with some variability in color of the metafemora. However, the accuracy of our morphological identifications is supported through an integrative taxonomic approach combining data from COI barcodes and morphometrics.

To assist future research of world *Asaphes*, we summarize the 13 described species with known distribution and habitat, and deposition of type material (Table 6). Based on our field studies and reports by Kamijo and Takada (1973) and Gibson and Vikberg (1998), it is highly likely that *Asaphes* mostly inhabit herbaceous areas such as cultivated fields, meadows, and potato fields. Interestingly, *Medicagosativa* (Fabaceae) was found in all our collecting sites of *A.vulgaris*. Therefore, we consider *A.vulgaris* to be most likely associated with *M.sativa*. Considering that *M.sativa* is an important economic green plant in Xinjiang and *A.vulgaris* is a hyperparasitoid, our results also indicate that it is harmful.

**Table 6.** Described species of *Asaphes* with known distribution and habitat, and deposition of type material.

Species	Distribution	Habitat	Deposition of holotype or lectotype	References
*A.brevipetiolatus*	Nearctic, Finland	subalpine meadow, mix conifer forest	CNC	Gibson and Vikberg 1998
*A.californicus*	Nearctic, Neotropical, China (Xinjiang, new record)	clover field, weed land, *Calamagrostispseudophragmites* (Poales: Poaceae)	USNM	Gibson and Vikberg 1998
*A.ecarinatus*	Yemen	unknown	DZUC	Narendran and van Harten 2007
*A.fuyunis*	China (Xinjiang)	miscellaneous grassland	ICXU	–
*A.globularis*	China (Tibet)	unknown	IZCAS	Xiao and Huang 2000
*A.hirsutus*	Nearctic, Mexico, western Palearctic	potato field, Ericaceae (Ericales), Boreal forest	CNC	Gibson and Vikberg 1998
*A.oculi*	China (Yunnan, Hebei)	unknown	IZCAS	Xiao and Huang 2000
*A.petiolatus*	Nearctic, western Palearctic	*Piceaglauca* (Pinales: Pinaceae)	MZLU	Gibson and Vikberg 1998
*A.pubescens*	Japan	shrubs, orchards, gardens, Coniferous woods, Deciduous woods, mixed woods	unknown	Kamijo and Takada 1973
*A.siciformis*	China (Yunnan, Sichuan, Hebei)	unknown	IZCAS	Xiao and Huang 2000
*A.suspensus*	Nearctic, Palearctic	cultivated fields, meadows, road side shrubs, orchards, gardens, coniferous woods, deciduous woods, mixed woods	HOPE	Kamijo and Takada 1973; Gibson and Vikberg 1998
*A.umbilicalis*	China (Jilin)	unknown	IZCAS	Xiao and Huang 2000
*A.vulgaris*	cosmopolitan	potato field	NHMUK	Gibson and Vikberg 1998; Xiao and Huang 2000

## Supplementary Material

XML Treatment for Asaphes

XML Treatment for Asaphes californicus

XML Treatment for Asaphes fuyunis

XML Treatment for Asaphes suspensus

XML Treatment for Asaphes vulgaris

## ﻿Additional information

### Author ORCIDs

Tong-You Zhang https://orcid.org/0009-0000-5425-3765

Gary A. P. Gibson https://orcid.org/0000-0002-8161-7445

Shi-Lei Shan https://orcid.org/0009-0002-0988-479X

Hui Xiao https://orcid.org/0000-0002-8979-3793

### Data availability

All of the data that support the findings of this study are available in the main text.
